# Endourologic strategies for a minimally invasive management of urinary tract stones in patients with urinary diversion

**DOI:** 10.1590/S1677-5538.IBJU.2017.0431

**Published:** 2018

**Authors:** FangLing Zhong, Gurioli Alberto, GuangMing Chen, Wei Zhu, FuCai Tang, Guohua Zeng, Ming Lei

**Affiliations:** 1 Department of Urology, Minimally Invasive Surgery Center, the First Affiliated Hospital of Guangzhou Medical University, Guangdong Key Laboratory of Urology, Guangzhou, China; 2 Department of Urology, Turin University of Studies, Turin, Italy

**Keywords:** Urinary Calculi, Urinary Diversion, Cystectomy

## Abstract

**Objective:**

To present our experience in minimally invasive management of urinary tract stones in patients with urinary diversion.

**Materials and Methods:**

We retrospectively reviewed 26 patients with urinary tract stones after cystectomy and urinary diversion. The types of urinary diversion were ileal conduit, colon conduit, ileal orthotopic neobladder in 19, 4, and 3 patients, respectively. At postoperative days 2, a plain KUB and urinary ultrasonography were performed in order to assess stone fragmentation or hydronephrosis. According to postoperative imaging, stone free rate (SFR) was defined as complete absence of fragments or residual stones less than 4mm.

**Results:**

19 patients were treated with minimally invasive percutaneous lithotripsy (MPCNL) and 2 patients required second-look MPCNL. Anterograde flexible ureteroscopy was performed in 2 patients, while in 2 patients a combined anterograde and retrograde approach was required. Three reservoir stones were treated by transurethral neo-bladder lithotripsy. Postoperative significant complications occurred in 2 patients (7.7%). The highest percentage of stone composition was struvite, as a result of chronic urinary tract infection (UTI). SFR was 88.5% (23 of 26).

**Conclusions:**

Our experience showed that MPCNL is a safe and effective treatment modality with little morbidity for renal and upper ureteral stones in patients with urinary diversion. For middle and lower ureteral stones, an anterograde approach could be also considered as a first line treatment, but a combined anterograde and retrograde approach was required when the anterograde access alone cannot provide acceptable results.

## INTRODUCTION

Radical cystectomy followed by urinary diversion is the treatment of choice for muscle invasive bladder cancer (1, 2). Patients with urinary diversion are highly predisposed to uretero-intestinal anastomotic strictures and urolithiasis as refluxing urine and pouch stasis may contribute to an increased risk for stone formation. Consequently, stone formation rate ranges from 9.0% to 26.5%, in this category of patients (3-5).

Urologists can be technically challenging for tissue adhesion after intestinal bladder reconstruction, ureteral anastomotic stricture and changes in patient health status. The management of urinary tract stones in patients with urinary diversion are varied, including PCNL, SWL, percutaneous based anterograde ureteroscopy, anterograde-retrograde combined ureteroscopy and even open approach (6, 7). Obviously, many factors must be considered in the treatment modality selection, such as stone size, stone location, diversion type, patient fitness and surgeon experience (8).

The objective of the present study is to review our experience, and assess the safety and efficacy of minimally invasive management of urinary tract stones in 26 patients with urinary diversion after cystectomy.

## MATERIALS AND METHODS

We retrospectively reviewed 26 patients with urinary tract stones who previously underwent cystectomy and urinary diversion. Any endourological procedure was performed at the Urology Department of the First Affiliated Hospital of Guangzhou Medical University between January 2011 and December 2016. Mean patient age was 63.12±10.01 years (range 40 to 78). Median interval between urinary diversion and stone management was 3.82 years (range 4 months to 19 years). Diversion type and patient's demographics are summarized in Table-1.

**Table 1:** Diversion type and Patients’ demographics (n=26).

Variable		Patients (%)
**Diversion type**		
	Ileal conduit (Bricker)	19 (73.1)
	Colon conduit	4 (15.4)
	Ileal orthotopic neobladder	3 (11.5)
**Sex**		
	Male	24 (92.3)
	Female	2 (7.7)
**Comorbidities**		
	Hypertension	8(30.8)
	Diabetes mellitus	5 (19.2)
**Presentation**		
	Hydronephrosis	11 (42.3)
	Urinary tract infection	11 (42.3)
	Flank pain	9 (34.6)
	Asymptomatic	6 (23.1)
	Fever >38°C	5 (19.2)
	Hematuria	2 (7.7)

The preoperative evaluation included medical history, physical examination, complete blood count, serum creatinine, urinary analysis, midstream urine culture and sensitivity test, coagulation profiles, ultrasonography, abdominal plain X-ray film of kidney, ureter, and bladder (KUB). In addition, non-contrast computed tomography (CT) was examined for evaluating stone characteristics. Intravenous urography (IVU) was performed in case of serum creatinine ≤150μmol/L. According to preoperative imaging, the stone size was noted by measuring the largest diameter. Patients with positive urine culture were managed by specific antibiotics and all patients routinely received prophylactic antibiotic before endourological treatment.

Furthermore, stones characteristics, intraoperative factors (operative time, complications) and postoperative results (SFR, complications, blood transfusion, and hospital stay) were recorded. SFR was defined as complete stone clearance or residual fragments less than 4mm at postoperative imaging.

## SURGICAL TECHNIQUE

### Kidney and upper ureter stones

The detailed technique of our MPCNL has been detailed in previous publications (9, 10). Nineteen prone MPCNL were performed under general anesthesia. Percutaneous access was obtained using ultrasonography or C-arm fluoroscopic guidance or a combined approach. We routinely started with ultrasonography guidance. An 18-gauge coaxial needle was used for renal puncture. The tract was dilated serially from 8 to a maximum 16-18F with a fascial dilator and a matched peel-away sheath was subsequently placed as the percutaneous access port. Kidney and upper ureteral stones were fragmented by pneumatic lithotripsy or holmium: YAG laser lithotripsy under an 8/9.8Fr semi-rigid ureteroscope (Richard Wolf, Germany). The large fragments were extracted with a forceps, and the small ones were flushed out with pulse perfusion pump.

### Middle and lower ureter stones

Pure retrograde ureteroscopy was firstly attempted in 4 patient, but all failed due to difficulty in entering the uretero-enteric anastomosis. Then, the patients were placed into the prone position and an anterograde flexible ureteroscopy (Olympus P5, Japan) was performed through a percutaneous access. A guidewire consequently allowed a retrograde approach. Stones were fragmented by YAG laser lithotripsy. Stone fragments were extracted using nitinol stone basket.

### Reservoir stones

Three patients were treated by transurethral neo-bladder lithotripsy. Ultrasonography was required to detect residual stones status during the operation. At the end of procedure, a 16Fr Foley catheter was left in place.

At the completion of procedure, a 4-6Fr double-J stent and a 18Fr nephrostomy tube were placed. Plain KUB and urinary ultrasonography at postoperative days 2 excluded hydronephrosis and assessed the SFR. In case of large residual fragments, a second-look MPCNL procedure was performed at least 3-5 days later. The nephrostomy tubes were removed in 3-4 days if stone free or with residual stone of <4mm. The double-J stent was removed 2-4 weeks after surgery. Postoperative follow-up including KUB and/or ultrasonography was usually scheduled at 3 months.

## RESULTS

Mean stone width was 1.56±0.69cm (range 0.3-3.4) while mean stone length was 2.13±1.25cm (range 0.6-4.0). The largest stone was over 4.0cm in diameter. Stone criteria and treatment results are summarized in Table-2.

**Table 2:** Stone criteria and treatment results (n=26).

Variable		Value (%)
**Stone side**		
	Right	14 (53.8)
	Left	12 (46.2)
**Stone location**		
	Kidney	17 (65.4)
	Upper ureter	2 (7.7)
	Middle ureter	3 (11.5)
	Lower ureter	1 (3.8)
	Reservoir	3 (11.5)
**Stone composition**		
	Struvite	16 (61.5)
	Calcium oxalate	7 (26.9)
	Urine acid	3 (11.5)
**Intervention received**		
	MPCNL	19 (73.1)
	Anterograde flexible	2 (7.7)
	ureteroscopy	
	Combined anterograde and	2 (7.7)
	retrograde approach	
	Transurethral neo-bladder	3 (11.5)
	lithotripsy	
Operation duration (min.)		69.42±26.01 (30-120)
Hospital stay (days)		8.50±3.06 (4-15)
**Complications**		
	Grade I (Fever)	2 (7.7)
Stone-free rate (SFR)		23 (88.5)

In our current series, 19 patients with renal and upper ureteral stones were treated by MPCNL. A ultra-sonographic guidance was used in 12 procedures while a combined ultra-sonographic and fluoroscopic approach was preferred in 7 patients. Immediate SFR was obtained in 15 (78.9%) patients after MPCNL. 2 patients required a second-look MPCNL to clear residual fragments during the same hospitalization. while 2 patients which a 6mm and 7mm residual fragment located in the lower calyceal with no hydronephrosis only needed conservative treatment. Thus, the SFR of MPCNL in these patients was 89.5% (17 of 19).

For 3 middle and 1 lower ureteral stones, two middle ureteral stones patients were treated with anterograde flexible ureteroscopy, using YAG laser lithotripsy and stone basketing. In the other 2 case, the anterograde approach could not reach the lower ureter because of severe ureteral angulation, consequently a percutaneous access was needed to advance a guidewire down to the neobladder and then a retrograde flexible ureteroscopy was performed. No residual stones were present in these 4 patients. One patient had 6mm residual stone in neo-bladder, they received conservative watching treatment.

Five patients additionally underwent incision and balloon dilation for combined ureteral obstruction and/or stricture. No intraoperative complication was registered in all patients. Postoperative significant complications were classified using the Clavien grading system (11). Two (7.7%) patients had fever (>38.5, Grade I), which was successfully cured by appropriate antipyretic. To sum up, the overall SFR was 88.5% (23 of 26 patients). After 24 months of follow-up, stone recurrence occurred in 4 patients (15.4%) (4/26): 2 patient were managed by conservative observation, while 2 needed MPCNL. Three patients presented uretero-intestinal stricture and persistent hydronephrosis, so double-J stents have been regularly replaced.

## DISCUSSION

With the rising prevalence of bladder cancer, many different forms of urinary diversions have been developed (2, 12). Patients with urinary diversion have to face with an increased risk of long-term complications, including stone formation and recurrence.

Preoperative hydronephrosis was observed in 11 patients (42.3%), as a result of uretero-enteric anastomotic stricture and/or upper tract stones. In the present study, after successful stone treatment and incision of the stricture, hydronephrosis disappeared after 3 months of follow-up. Hyams et al. (13) reported that short strictures (length<1cm) were successfully accessed and treated with balloon dilation and/ or endo-ureterotomy. Regular follow-up is of quite importance for early detection and intervention of stone recurrence and ureteral stricture disease in patients after urinary diversion.

In our study, stone analysis was performed with infrared spectroscopy. Stone composition included: struvite (61.5%), calcium oxalate (26.9%) and uric acid (11.5%). The highest percentage of struvite stones in patients with urinary diversion can be the result of chronic urinary tract infection (UTI) as reported by Hertzig (14) and El-nahas (15). Metabolic factors, including metabolic acidosis, hypercalciuria, hyperoxaluria and hypocitraturia play important roles in struvite stone development (5). Comprehensive management of struvite stone should consider perioperative antibiothic therapy and it should be prolonged postoperatively in the presence of significant residual fragments (16).

Several studies supported the use of SWL in treating upper tract stones after cystectomy and urinary diversion. El-Assmy et al. (6) reported a 81.5% (22 of 27) overall success rate of SWL monotherapy in the treatment of upper urinary tract in this category of patients. On the other hand, Seth et al. (12) reported higher rates of complications (such as steinstrasse) after SWL rather than other endourologic procedures. Furthermore, retreatment rates of SWL are considerably high (17).

Pure retrograde access may be difficult in localizing the ureteral orifice and is considered a big challenge for endourologist even in experienced centers. Consequently, an anterograde approach should be considered after retrograde failure for middle and lower ureter stones. Moreover, antegrade ureteroscopy through the established tract was used to advance a guidewire. As soon as access was obtained, retrograde management provided us to perform all required diagnosis and treatment. The adjunct of flexible nephroscopy and/or ureteroscopy to standard PCNL has potential benefits, including reducing need for multiple accesses and improving both efficacy and safety of PCNL (18).

PCNL is the gold-standard for patients with large and complex upper urinary tract stones. In the past, standard PCNL was usually performed through a 24 to 30F access tract. Recently, MPCNL is a modified standard PCNL technique using a miniaturized instrumentation through a smaller nephrostomy tract (14-18F). In our center, we have been using MPCNL for the treatment of complex upper urinary calculi over 20 years and more than 10.000 cases have been successfully accomplished (9, 10). Our study presented that the SFR of MPCNL was 89.5% (17 of 19 patients). In our experience, the smaller renal access is less damaging for the renal parenchyma and this may contribute to reduce procedure-related complications. According to a meta-analysis study by Zhu et al. (19), it has been reported that MPCNL possess an apparent advantage in less bleeding, fewer transfusion rate, less pain and shorter hospital stay when compared to standard PCNL. For all these reasons, MPCNL is particularly useful in removing upper tract stones in view of its high SFR, and this procedure is particularly indicated in frail patients with urinary diversion.

Our study has some limitations. It is a retrospective study with an inherent bias in management selection and follow-up. A larger series study associated with metabolic evaluation could help to better explore this problem in the future.

## CONCLUSIONS

Our experience showed that MPCNL is a safe and effective treatment modality with minor morbidity for kidney and upper ureter stones in patients with urinary diversion. For middle and lower ureteral stones, anterograde approach could be considered as first line treatment, but a combined anterograde and retrograde approach is required when the anterograde access alone cannot provide acceptable results.
